# More than a biomarker: could language be a biosocial marker of psychosis?

**DOI:** 10.1038/s41537-021-00172-1

**Published:** 2021-08-31

**Authors:** Lena Palaniyappan

**Affiliations:** 1 Department of Psychiatry, Schulich School of Medicine and Dentistry, University of Western Ontario, London, ON Canada; 2 Department of Medical Biophysics, Schulich School of Medicine and Dentistry, University of Western Ontario, London, ON Canada; 3 Robarts Research Institute, University of Western Ontario, London, ON Canada; 4 Lawson Health Research Institute, London, ON Canada

**Keywords:** Biomarkers, Psychosis

## Abstract

Automated extraction of quantitative linguistic features has the potential to predict objectively the onset and progression of psychosis. These linguistic variables are often considered to be biomarkers, with a large emphasis placed on the pathological aberrations in the biological processes that underwrite the faculty of language in psychosis. This perspective offers a reminder that human language is primarily a social device that is biologically implemented. As such, linguistic aberrations in patients with psychosis reflect both social and biological processes affecting an individual. Failure to consider the sociolinguistic aspects of NLP measures will limit their usefulness as digital tools in clinical settings. In the context of psychosis, considering language as a biosocial marker could lead to less biased and more accessible tools for patient-specific predictions in the clinic.

“I have resisted the term sociolinguistics for many years, since it implies that there can be a successful linguistic theory or practice which is not social”^1^

## Computational linguistics in psychosis

In recent times, a surge of methodological advances in sampling human discourse has brought the spotlight back on the phenomenon variously known as ‘formal thought disorder’ (FTD), ‘speech disorder’ or ‘communication disturbances’ in schizophrenia^2–5^. The emphasis on objective measurement of speech in real-world settings using automated assessment is not new^6^. But the improved access to normative corpora for analysis, the corpus-independent graph metrics and the emergence of ambulatory approaches for speech capture that aid Artificial Intelligence based learning systems have improved scalability, and provided the much needed momentum to this field of inquiry. Several predictive as well as mechanistic studies have been published in npj Schizophrenia^7–11^ in recent times, with excellent state-of-art appraisals of computational linguistics found elsewhere^2,4,5,12^. The assessment of thought disorder has extended from being a qualitative assessment using FTD scales to a more quantitative determination based on automated measures.

To date, the emphasis of quantitative speech studies has been a utilitarian one. This involves examining language as a biomarker of pathogenic neural processes of schizophrenia, and leveraging this biomarker for diagnostic and prognostic purposes. In fact, natural language processing (NLP) measures, often digitally acquired, have been evaluated as biomarkers of risk^7,13–15^, diagnosis/prognosis^8,16^, and to study pharmacological effects^10^ in recent times. In this broad context of use, NLP measures are treated as many other biomarkers investigated in psychiatry^17^.

## Sociomarkers, biomarkers and biosocial markers

The term ‘sociomarkers’ is sometimes used for objective characteristics that mark a social condition or process that an individual has experienced or currently embedded in. For example, neighbourhood housing quality could be a social marker to predict hospital visits in children with asthma^18^. Some sociomarkers (e.g., homelessness) can also be used as a proxy endpoint for systems-interventions in multidisciplinary mental health settings. However, variations in these systems-level sociomarkers (e.g., homelessness) are often not attributable to changes in human biology. In contrast, quantity and quality of speech (or written language) can be manipulated via several biological interventions (e.g., ketamine infusion^19^, neuromodulation^20^, vascular insults etc. to name a few). These linguistic measures are not only objectively quantifiable but also change with disease processes; thus, linguistic markers satisfy the broadly accepted criteria for a biomarker^21^.

While several biomarkers are affected by social factors, their quantification process itself is unlikely to be socially influenced. The structure of human language is heavily influenced by sociocultural^22^ and contextual factors^23,24^, in ways that are much deeper than the effect of these factors on conventional tissue-based biomarkers. First, our everyday speech is replete with markers of our present and past social states^25^ e.g., schooling, level of one’s education, current social network to name a few. What we say, and how we say it, depends on the immediate social context in which a speech event is embedded. The social and professional status of the speakers, in-group vs. out-group differences, class, ethnicity, age, gender as well as the social relatedness^23^ provide this social context. Quantitative linguistic markers such as lexical diversity are strongly correlated with socioeconomic markers such as neighbourhood income levels and density^26^. More relevant to the study of psychosis, is the large body of literature supporting the effect of early life socioeconomic status on quantitative speech markers (often referred to as the “million word-gap”^27,28^). Besides lexical diversity, social determinants such as social class affect syntactic complexity^29^, while parental education status relates to frequency of certain parts of speech in adult speakers. For example, patients with schizophrenia born to better educated parents use more conjunction, less personal and first person singular pronouns^30^. Graph-based linguistic markers, considered to be predictive of diagnostic outcomes in psychosis^8,15^, relate to family income and more strongly, to the number of years of school education^31^. Features of formal thought disorder are pronounced in maltreated children^32^; in terms of quantitative markers, the experience of institutional care has profound effects on lexical diversity and mean length of utterances during childhood, as demonstrated by the remarkable randomized trial of foster care vs. institutional care in Bucharest^33,34^. Such quantitative differences in early lexical development can affect later acquisition of syntax, and the processing dynamics in adult speakers^35^ (also see^36^).

Linguistic structure that contributes to the various quantitative speech markers used in the study of psychosis, varies with the language being studied. Parts of speech that are reportedly abnormal in schizophrenia, are not universally present across languages. For example, in psychosis, formal thought disorder in English speakers relates to excess use of pronouns^37,38^ and discourse connectives^39^, but reduced use of articles and prepositions relative to other function words^40,41^. In particular, first person singular pronouns (which by definition excludes referring to the listener) are increased^38,42^ while first person plural pronouns (which may or may not exclude the listener) are reduced in the presence of psychosis^36^. Such a pattern cannot be gleaned if we examine patient speech transcripts in a different language, Tamil, for example. Tamil, one of the few living classical languages, splits the first person plural to inclusive and exclusive versions, second person pronouns to singular and plural versions, has no articles, uses suffixes rather than unbounded discourse connectives, and has no prepositions but only postpositions^43,44^. Bilingual speakers whose first language (L1) structure varies from English (L2), often continue to make subtle structural errors^45,46^ and display altered semantic coherence^47,48^. Even within a given language, the performance of NLP algorithms can vary with the dialect being spoken^49^. This increases the risk of some speakers being mis-classified as being deviant from other healthy subjects, if such speech markers are used without due consideration of sociolinguistic differences. Thus, speech markers of psychosis derived from one language, or one dialect, may not always perform well for a patient speaking a different dialect, or has a different L1. This introduces another layer of socio-developmental variability with no biological causal basis.

In summary, the early life language environment is likely to have a critical effect on several quantitative speech markers measured in adult speakers. Linguistic measures are not merely markers of a biological state; they are also ‘fossils’ of one’s social circumstances. Thus, language is best considered as a biosocial marker. To this end, a significant portion of inter-individual variance in linguistic structure among patients with psychosis could also relate to social factors. This speculation, based on the data from non-psychotic individuals reviewed above, is indirectly supported by several empirical observations linking qualitative measures of thought disorder with social factors in psychosis.

## Social determinants of ‘formal thought disorder’

Language difficulties appear long before a clinical or preclinical state can be defined in psychosis^50,51^. Specific qualitative features of formal thought disorder that are apparent after the onset of psychosis relate to various social factors such as social class, educational exposure, immigrant status and social isolation in patient samples. When studying the generalisability of Thought Disorder Index (TDI), Haimo and Holzman observed that lower social class related to higher TD scores in healthy subjects, but to lower TD scores among patients with schizophrenia^52^, highlighting the differential role that social class can play in the assessment of TD. Poverty of thought is more pronounced in the less educated patients^39^, and those with parents from lower socioeconomic status (especially in female patients^53^). More recently, Nogueira and colleagues reported a relative excess of formal thought disorder among individuals with familial migration history at ultra-high risk of psychosis, though this study was limited by its sample size^54^. Nevertheless, these findings resonate with Berg and colleagues^55^ who showed in a large Norwegian sample (*n* = 1081) that while natives and immigrants had mostly similar symptom profile, the most prominent difference in symptom dimensions, especially in visible minorities, related to disorganisation factor of PANSS (especially, difficulties in abstract thinking). Social isolation is associated with disorganised thinking more than any other positive symptoms of psychosis^56,57^. A reduction in exposure to social dialogue and feedback due to extended social isolation that occurs in the face of immigration, frequent school dropouts^58^, institutionalisation may affect the ability of perspective-taking, increasing the risk of speech disturbances^59^.

While some of the above associations can result from a reverse causality e.g., poverty of thought contributing to educational failure and disorganisation contributing to social isolation, other social factors precede the onset of language disturbances and cannot be assumed to result from a patient’s TD per se (e.g., parental immigration, parental socioeconomic status, minority social status). Taken together, a broad range of social states affect the degree of language disturbances in psychosis, though most of the existing evidence comes from examinations of qualitative deviations (i.e., formal thought disorders). Insofar as the quantitative NLP markers relate to TD, they may also be altered by the several interacting social factors that influence TD; this requires systematic evaluation in future studies.

## Is language the only marker to be socially influenced?

Given the promising translational value of computational linguistic markers in the era of digital health, this Perspective focuses specifically on language. Nevertheless, many putative biomarkers are likely influenced by social factors; one may argue that linguistic markers are not exceptional in this regard. For example, social adversities affect endocrine, metabolic and neural biomarkers^60^, though some markers are affected more by social factors than the other^61,62^. This raises the question whether linguistic markers of disease states are more ‘socioplastic’ than non-linguistic biomarkers. At present, we do not know if social factors affect linguistic markers more than they affect the other putative biomarkers of psychosis (e.g., hippocampal volume, cortical gyrification). We also do not know if social factors explain proportionately more variance than biological factors for putative linguistic markers in psychosis. Given the lack of data from clinical samples, it is worth considering certain factors that indirectly imply the relative importance of social factors on linguistic markers.

Linguistic markers (both verbal and nonverbal) carry explicit information that can identify individuals to the social groups or places to which they belong^63^. Some aspects of language can even be altered intentionally in response to one’s social needs (e.g., code-switching and thus the choice of words and syntax^64^). In addition, linguistic markers can also be affected by the context in which the measurement is undertaken (e.g., the formality and the familiarity of the receiver affects markers of linguistic sentiment^65^), supporting the argument that participant-level speech markers “on closer examination prove to be markers not of participant per se, but of participant in a particular situation”^23^. These social contributors *(identity*, *necessity, familiarity* and *situationality*) are unique to language and cannot be significant influences on illness-related biomarkers such as brain volume or gyrification.

In the wake of the circumstantial evidence reviewed here, the influence of social factors on linguistic markers of psychosis requires further scrutiny. Irrespective of the eventual influence apportioned to biological vs. social factors, neglecting one aspect in favour of the other risks oversimplification and may be detrimental to the development of computational linguistic applications in psychosis.

## Implications and future directions

Speech is an easily accessible marker to monitor psychosis; with NLP approaches, the highly desired objectivity in speech analysis now appears achievable. But this objectivity does not confer a complete freedom from social influences to NLP measures of language in psychosis. For example, Hitczenko and colleagues recently observed that sociodemographic differences in certain NLP measures of coherence are larger than the differences related to psychosis at-risk state^66^, though this study only included a small number of subjects. Such outcomes may be the result of inherent biases in the corpora used for word embedding models as discussed elsewhere^67,68^; at present, we do not know the degree to which such socially driven biases contribute to prediction accuracies of NLP algorithms in clinical cohorts with psychosis. The relative influence of social factors is unlikely to be uniform across the various quantitative speech measures. NLP based predictive studies in psychosis need to urgently consider the influence of factors such as parental/individual socioeconomic status, neighbourhood deprivation levels, education or immigration status on the various structural speech markers that are under scrutiny^12^. (See Table 1)

**Table 1:** Social factors with reported influence on quantitative speech markers.

Speech markers relevant to psychosis	Non-biological (social) factors with demonstrable influence in healthy subjects
Graph connectivity	Family income
	Years of education
Length of utterance	Institutionalization
Lexical diversity	Parental socioeconomic status
	Neighbourhood
	Institutionalization
Parts of speech (function words)	Parental education
	Bilingualism
	Dialect variations
Pauses and prosody	Binary entity described as race^a^
	Dialect variations
Semantic coherence	Binary entity described as race^b^
Semantic density	Bilingualism
Syntactic complexity	Social class

All listed speech markers are identified as promising candidates for computational linguistic analysis in psychosis (see Hitczenko and colleagues^12^). Individual studies with supporting evidence are discussed in the text. Most of the included studies were not specifically powered to examine the reported associations; the associations listed in this table are best considered as signals that require further systematic evaluation and not as conclusive links.

^a^Race was evaluated as a binary variable (Black or African-American/White)^66,67^, without a multidimensional assessment of underlying components^75^.

As specific applications of NLP markers for psychosis become more clear, rigorous tests against evidentiary criteria^69^ will be required to enable clinical use. At this stage, the performance of NLP assays (i.e., predictive consistency, dose-response relationships, generalizability) and their clinical interpretability will depend on the effects of social factors on these assays. For example, if transition to psychosis is more prevalent among individuals that dropped out of school, and if such individuals also have lower semantic coherence (a NLP measure), the predictive performance of semantic coherence for transition may only reflect the contribution of poor school retention. When applied to a social milieu with uniformly better school retention, the algorithm will have a poor yield, as the variance in semantic coherence will be now reduced. This does not necessarily diminish the mechanistic importance of the language disturbance per se, as one can conceive a causal link between linguistic issues and school dropout, and both being predictive of psychosis. Nevertheless, the relative utility of this NLP measure in prognostic prediction is likely to be reduced if its relationship with readily measurable and differentially distributed social determinants is not fully considered. Besides analytical and clinical validations^70^, the widespread use of digital language-based biomarkers in psychosis requires a careful evaluation of the social determinants of linguistic aberrations highlighted here.

The view of NLP outputs as biosocial markers rather than biomarkers has two important implications. First, this reminds us that the algorithmic bias, defined as the amplification of existing social inequities when employing Artificial Intelligence algorithms^71^, is a critical issue when using linguistic data. Second, the biosocial emphasis highlights the need for social diversity in the participant recruitment to obtain meaningful predictive values^72^. If unattended, these issues may ordain the NLP based algorithms to a limited utility (poor incremental value). In an extreme case scenario, such NLP-based predictions may paradoxically be less useful for the underrepresented groups, for whom prognostic prediction is likely to be most valuable.

An ideal NLP-based algorithm should operate in an unbiased manner, have equitable performance across different health systems, and provide actionable results. We require certain interventions to achieve this goal. NLP studies in psychosis must seek diverse samples and report social indicators with diligent detail. Besides predictive accuracy, algorithms must be examined for contextual specificity e.g., test performance at different social class or immigration strata. Generalisability across social groups/contexts should not be automatically assumed before such efforts are undertaken. With context-specific performance metrics, we will be able to build clinically meaningful counterfactual explanations^73^ for NLP-based test results. Third, attempts to quantify the degree of social influence on the various quantitative linguistic markers is required to build multilevel models without redundancy. Building a large corpus of multilingual ‘benchmark dataset’^74^ for psychosis that can capture sufficient ethnic, cultural, social, economic, educational and lifestyle differences is a crucial step in this regard.
